# Effects of feed intensity and breed on postpartum blood metabolites

**DOI:** 10.1186/1751-0147-57-S1-O12

**Published:** 2015-09-25

**Authors:** Theodoros Ntallaris, Patrice Humblot, Ylva Sjunnesson, Renée Båge, Joëlle Dupont, Britt Berglund

**Affiliations:** 1 Department of Clinical Sciences, Swedish University of Agricultural Sciences, Uppsala, Sweden; 2 INRA PRC, Tours, France; 3 Department of Animal Breeding and Genetics, Swedish University of Agricultural Sciences, Uppsala, Sweden

## Introduction

In dairy cows, postpartum negative energy balance impairs fertility.

## Objective

The aim of this study was to investigate the influence of high and low feed intensity starting one month before first calving on blood metabolites of energy balance in Holstein (H, n=14) and Swedish Red dairy breed (SRB, n=14) kept in a loose housing system.

## Material and methods

The control group (C, n=12) was fed a diet targeting high production (35kg/day Energy-Corrected Milk, ECM). The lower feeding intensity (L, n=12) was achieved by giving -50% concentrate to target 25kg/day ECM. Blood was sampled every 2 weeks until 2 months postpartum and then once a month until Day 120. Plasma was kept frozen until analysis for glucose, insulin and non-esterified fatty acids (NEFA) concentrations. Data were analyzed with Mixed linear models.

## Results

For glucose, effects of breed (p<0.01) and time (p=0.01) were observed. Postpartum glucose levels were constantly higher in SRB when compared to H cows (56.7 ±1.9 vs 49.9 ±2.0 mg/dl). For insulin, there was an interaction between diet and time (p<0.03); concentrations being higher in C than in the L group until 3 months and not different later. For NEFA's an effect of time was observed (p=0.001) and a tendency (p<0.10) for interaction between breed and diet. Holstein tended to have lower NEFA concentrations with the low diet whereas the inverse was observed for SRB.

## Concusion

These results indicate that feed intensity and breed influence energy mobilization. However, no relationships were found with reproductive variables.

## Funding

The study was financed by “PROLIFIC” EU grant N°311776.

